# Compositional and Bioactive Characterization of Industrial Fruit Wastes in Sri Lanka

**DOI:** 10.1111/1750-3841.70938

**Published:** 2026-02-18

**Authors:** Chamila Vinodanee Liyanage Jayasinghe, Jayasundara Mudiyanselage Lakshan Randika Bandara, Rankothge Chamodini Nisansala Thilakarathna

**Affiliations:** ^1^ Department of Food Science and Technology, Faculty of Livestock, Fisheries and Nutrition Wayamba University of Sri Lanka Makandura Sri Lanka; ^2^ School of Science Monash University Malaysia, Jalan Lagoon Selatan, Bandar Sunway Subang Jaya Selangor Malaysia; ^3^ Department of Food Science and Technology, Faculty of Applied Sciences Sabaragamuwa University of Sri Lanka Belihuloya Sri Lanka

**Keywords:** functional activity, industrial fruit processing waste, proximate and mineral composition

## Abstract

The potential of industrial fruit processing wastes for nutritional and bioactive applications was evaluated. A survey of four major fruit processors in Sri Lanka identified pineapple (*Ananas comosus*), mango (*Mangifera indica L*.), papaya (*Carica papaya*), and passion fruit (*Passiflora edulis*) as the most processed fruits, generating waste yields of 69.98%, 50.07%, 48.41%, and 61.21%, respectively, primarily as peels, seeds, and bagasse. Analysis of dehydrated wastes revealed crude protein (0.50–25.94%), crude fat (0.36%–28.16%), and crude fiber (12.97–43.10%). Essential minerals (K, Ca, Mg, Zn, Cu) were present, while toxic metals were mostly below permissible limits. Phytochemical evaluation showed total phenolic contents of 1.85–9.47 mg Gallic Acid Equivalents/g and flavonoids of 1.38–21.82 mg Rutin Equivalents/g. Total antioxidant capacity ranged from 1.85–25.54 mg Ascorbic Acid Equivalents/g, and DPPH radical scavenging activity (IC_50_) ranged from 0.53–54.18 mg Acetic Acid Equivalent/mL. Mango and papaya peels, along with mango seed kernels, exhibited the highest antioxidant activity, consistent with their phenolic and flavonoid levels. These results indicate that industrial fruit wastes are nutrient‐ and bioactive‐rich materials, offering significant potential for functional food and nutraceutical applications, contributing to waste valorization and sustainable food processing.

## 1 Introduction

Sri Lanka, located within the tropical climatic belt, is characterized by three major agro‐climatic zones: the wet, intermediate, and dry zones (World Bank, CIAT 2015). These diverse climatic conditions support the cultivation of a wide range of fruits and vegetables. Approximately 80 fruit varieties are produced in the country, with an annual production of nearly 900,000 metric tons supplying both the domestic and international markets (SLEDB 2016). Despite this production capacity, the Institute of Post‐Harvest Technology (IPHT) in Sri Lanka has estimated that 30%–40% of total fruit production is lost during pre‐ and post‐harvest handling (IPHT 2015). In addition to these losses, industrial fruit processing generates substantial quantities of waste that have not yet been systematically quantified or scientifically evaluated for potential utilization in Sri Lanka. The accumulation of fruit processing wastes poses significant economic, environmental, and public health concerns (Bakas 2010). Improper disposal contributes to odor generation, greenhouse gas emissions, disease transmission, and environmental degradation (Bharat and Sahoo 2016; Dhillon et al. 2013; Narashans et al. 2014). Reducing post‐harvest losses and promoting the effective utilization of fruit processing wastes are therefore essential for improving resource efficiency, addressing food insecurity, and mitigating environmental impacts (Lundqvist et al. 2008).

Globally, there is growing interest in recovering value‐added bioactive compounds from fruit processing by‐products, such as seeds, peels, and bagasse. These compounds, including dietary fiber, proteins, and antioxidants, have demonstrated potential applications in the food and nutraceutical industries. For example, papaya peel has been utilized as a flour ingredient and as a source of protease (Barretto et al. 2013; Chaiwut et al. 2010), while recent studies have demonstrated the extraction of pectin from banana peel for use as a functional fat replacer in bakery products (Ahsan et al. 2024). Mango seed kernels contain bioactive gallotannins with antimicrobial activity (Engels et al. 2009; da Silva et al. 2014), and fruit processing by‐products are generally recognized as rich sources of dietary fiber and antioxidants with potential applications in functional foods. Fruit wastes are also rich in polyphenols, minerals, and dietary fiber, which contribute to nutritional enrichment and the prevention of oxidative stress‐related diseases (Ismail et al. 2024; Silva et al. 2024). Recent studies highlight the innovative use of fruit waste in bakery products, such as banana and mango peels in biscuits and muffins (Ismail et al. 2024), and melon peel flour in biscuits and muffins with high fiber and phenolic content (Silva et al. 2024). These findings emphasize that fruit processing by‐products are a sustainable source of functional ingredients for healthier food products.

In Sri Lanka, fruit processing industries commonly utilize mango (*Mangifera indica* L.), papaya (*Carica papaya* L.), passion fruit (*Passiflora edulis* f. *flavicarpa* L.), pineapple (*Ananas comosus*), watermelon *(Citrullus lanatus*), ash gourd (*Benincasa hispida*), and wood apple (*Limonia acidissima*) for the production of juices, jams, pulps, cordials, and canned products. However, comprehensive quantification of processing wastes and detailed analysis of their bioactive compounds remain limited. Therefore, the present study was conducted to quantify the types and amounts of waste generated by selected fruit processing industries in Sri Lanka, to evaluate their nutritional and bioactive properties, and to explore potential avenues for value‐added utilization.

## 2 Materials and Methods

### 2.1 Chemicals

Ascorbic acid, rutin, gallic acid, Folin–Ciocalteu reagent, 1,1‐diphenyl‐2‐picrylhydrazyl (DPPH), ammonium molybdate, and methanol were purchased from Sigma‐Aldrich (St. Louis, MO, USA) through Analytical Instrument Pvt Ltd, Colombo, Sri Lanka. All other chemicals used in this study were of analytical grade.

### 2.2 Plant Materials

Fruit waste samples of eight types of fruits, that is; mango (*Mangifera indica* L.), papaya (*Carica papaya* L.), passion fruit (*Passiflora edulis* f. *flavicarpa* L.), pineapple (*Ananas comosus*), watermelon *(Citrullus lanatus*), lime (*Citrus aurantiifolia*), ash gourd (*Benincasa hispida*), and banana (*Musa spp*.), were collected from four leading fruit processing companies (coded as A, B, C, and D for confidentiality) located in the Western and NorthWestern Provinces in Sri Lanka. Mechanically separated fresh fruit wastes were collected as peel, while seeds and bagasse were taken after juice extraction, particularly in the case of pineapple.

### 2.3 Preparation of Dehydrated Fruit Waste Powder (DFWP)

Fresh fruit waste samples (peel, seed, and bagasse) were dried in a food dehydrator (32609, TSM, USA) at 45–50°C for 12 h. The dried samples were ground using a grinder (IS: 4250, Abans, India) and sieved through a mesh (< 425 µm) using a laboratory sieve. The resulting powders were packed in airtight brown glass bottles and stored at ‐20°C until use.

### 2.4 Identification and Quantification of Types of Fruit Waste

A survey was conducted among four major fruit processing companies (A, B, C, and D) in Sri Lanka to identify and quantify the amount of fruit processing waste. Company records, discussions with responsible personnel, and direct measurements by the research team were used to quantify the waste. During the survey period, the company was provided with plastic containers to collect each waste type separately, and the weights were recorded. When direct separation was not feasible, representative fruit samples (10–20 kg) were manually sorted, and the waste fractions were weighed. The waste percentage was calculated using Equation (1).

(1) $$\begin{array}{ccc} \mathrm{Waste}\% & = & \left[\mathrm{Fresh}\mathrm{weight}\mathrm{of}\mathrm{fruit}\mathrm{waste}/\mathrm{Fresh}\mathrm{weight}\mathrm{of}\mathrm{whole}\mathrm{fruit}\right]\\ & & \times \;100\% \end{array}$$

All measurements were performed in triplicate, and the results were expressed as mean values. Fruit waste was categorized into peel, seeds, rind, and bagasse according to the type of fruit.

### 2.5 Proximate Compositional Analysis of Fruit Waste Samples

#### 2.5.1 Moisture Content

The moisture content of DFWP was determined by the oven‐drying method, as described in AOAC 2000, 934.06. Pre‐weighed samples were dried in a hot air oven (Mermmet: OF‐22G, Korea) at 105°C for 3 h. The moisture content was calculated from weight loss.

#### 2.5.2 Protein Content

The crude protein content was determined using the micro Kjeldahl method (AOAC, 2000, 955.04). Sample digestion and distillation were performed using a Kjeldahl apparatus (4000629, J.P. Selecta, Spain). Protein content was calculated using a nitrogen conversion factor of 6.25 and expressed as a percentage of dry weight.

#### 2.5.3 Fat Content

Crude fat content was determined by Soxhlet extraction (AOAC, 2000). Approximately 3–5 g of dried sample was extracted using petroleum ether in a Soxhlet apparatus (00A‐56826, J.P. Selecta, Spain). Results were expressed on a dry weight basis.

#### 2.5.4 Fiber Content

The crude fiber content was determined using the Weende method (AOAC, 1999). Dried samples were digested using a fiber analyzer (00A‐619, J.P. Selecta, Spain) with boiling sulfuric acid, followed by sodium hydroxide. The residue was washed and dried in an oven (Memmert, OF‐22G, Korea) at 105 ± 1°C and then incinerated in a muffle furnace (Hobersal, Rex‐C700, Spain) at 550 ± 25°C. Crude fiber content was calculated based on weight differences.

#### 2.5.5 Ash Content

Ash content was determined by incinerating 5 g of DFWP in a muffle furnace (Hobersal, Rex‐C700, Spain) at 550°C for 4–5 h, until constant weight was achieved.

#### 2.5.6 Mineral and Heavy Metal Content

Mineral elements (Ca, Na, Mg, K, Zn, Cu) and heavy metals (As, Cd, Hg, Pb) were analyzed using inductively coupled plasma mass spectrometry (ICAP MSX, Thermo Fisher Scientific, USA). Digestion of DFWP was conducted using a microwave digester (CEM GmbH, MARS 6, USA) according to the AOAC method (2015.01). The digested samples were filtered (Whatman Grade 5, pore size 2.5 µm), diluted to 50 mL with deionized water, refiltered using syringe filters (25 mm PTFE 0.45 µm), centrifuged, and analyzed.

#### 2.5.7 Carbohydrate Content

The carbohydrate content was calculated by difference according to FAO (2003) using Equation (2).

(2) $$\begin{array}{ccc} \mathrm{Carbohydrate}\left(\%\right) & = & 100-\left(\mathrm{Total}\mathrm{fat}+\mathrm{Crude}\mathrm{protein}\right.\\ & & \left.+\;\mathrm{Crude}\mathrm{Fiber}+\mathrm{Total}\mathrm{ash}\right) \end{array}$$

### 2.6 Phytochemical Analysis

#### 2.6.1 Preparation of the Methanol Extract of the Fruit Peel

One gram of DFWP was mixed with 25 mL of methanol in a conical flask and extracted in a shaking water bath at room temperature (25°C) and 100 rpm for 3 h. Extracts were centrifuged at 5000 rpm for 20 min using a MiniSpin plus centrifuge (Eppendorf Type 22331, Germany). The supernatant was filtered (Whatman No. 1, UK), adjusted to 25 mL with methanol, transferred to brown glass bottles, and stored at −18°C until analysis.

#### 2.6.2 Total Phenolic Content

The total phenolic content was determined using the Folin‐Ciocalteu method (Singleton and Lamuela 1998) with some modifications. Briefly, 0.5 mL of the extract or gallic acid standard solution was mixed with 1 mL of Folin‐Ciocalteu reagent and incubated in the dark at room temperature for 10 min. Subsequently, 2 mL of 7% sodium carbonate was added, and the mixture was incubated in the dark at room temperature for 2 h. The absorbance was measured at 760 nm using a UV/VIS spectrometer (Optima, SP‐3000, Tokyo, Japan). A calibration curve was prepared using gallic acid at concentrations of 5–100 mg/mL (R^2^ = 0.9999). The results were expressed as mg of gallic acid equivalent (GAE) per g of dry weight.

#### 2.6.3 Total Flavanoid Content

The total flavonoid content was determined using the aluminum chloride colorimetric method (Zhishen et al. 1999), with minor modifications. Briefly, 0.5 mL of the methanolic extract or rutin standard solution was mixed with 3 mL of distilled water, followed by the addition of 0.3 mL of 5% NaNO_2_, and then incubated at 30°C for 5 min. Then, 0.3 mL of 10% AlCl_3_ was added and incubated for 6 min, followed by the addition of 2 mL of 1 M NaOH. The volume was adjusted to 10 mL with distilled water and mixed. The absorbance was measured at 510 nm using a UV‐VIS spectrometer (Optima, SP‐3000, Tokyo, Japan). A calibration curve was prepared using rutin at concentrations ranging from 5 to 100 mg/mL (R^2^ = 0.9958), and the results were expressed as mg rutin equivalents (RE) per g dry weight.

#### 2.6.4 DPPH Scavenging Activity

The free radical scavenging activity was determined using the DPPH assay according to Hatano et al. (1988) with minor modifications. An aliquot of 3 mL of freshly prepared DPPH solution was mixed with 1 mL of the sample or acetic acid standard solution at various concentrations, and the mixture was vortexed for approximately 5 seconds. The reaction mixtures were incubated in the dark at room temperature for 30 min, and absorbance was measured at 517 nm using a UV‐Vis spectrometer (Optima, SP‐3000, Tokyo, Japan). Methanol was used as the blank, and the methanolic DPPH solution served as the control. A standard calibration curve was prepared using acetic acid at concentrations ranging from 5 to 50 mg/mL (R^2^ = 0.9972). The inhibition % was calculated from Equation 3 and plotted against concentration for each sample, and the IC_50_ values were determined as the concentration required to inhibit 50% of DPPH radicals.

(3) $$\begin{array}{ccc} \mathrm{Inhibition}\left(\%\right) & = & \left[\left(\mathrm{Absorbance}\;\mathrm{of}\;\mathrm{control}-\mathrm{Absorbance}\;\mathrm{of}\right.\right.\\ & & \left.\left.\mathrm{extract}\right)/\mathrm{Absorbance}\;\mathrm{of}\;\mathrm{control}\right]\times 100 \end{array}$$

#### 2.6.5 Total Antioxidant Activity

The total antioxidant capacity was determined according to the method described by Prieto et al. (1999) with slight modification. Briefly, 0.3 mL of methanolic extract or ascorbic acid standard solution was mixed with 3 mL of reagent solution (0.6 M sulfuric acid, 28 mM sodium phosphate, and 4 mM ammonium molybdate). The tubes were capped and incubated in a thermal block at 95°C for 90 min. After cooling to room temperature, the absorbance was measured at 695 nm against a blank. A calibration curve was prepared using ascorbic acid at concentrations ranging from 5 to 100 mg/mL (R^2^ = 0.9967), and the results were expressed as mg ascorbic acid equivalent (AAE) per g of dry weight.

### 2.7 Statistical Analysis

The statistical analysis of data was carried out using one‐way analysis of variance (ANOVA) with significant differences between means at *p* < 0.05, and all data were exhibited as the mean with standard deviation of triplicate determinations.

## 3 Results and Discussion

### 3.1 Quantification of Industrial Fruit Wastes

Information on fruit waste generation was collected from four leading fruit processing companies in Sri Lanka (Table 1). Pineapple, mango, papaya, and passion fruit were the most extensively processed fruits during the survey period, while wood apple, ash melon, and lime were processed to a lesser extent. Fruit waste primarily consisted of peels, seeds, bagasse, and crowns, depending on the fruit type and processing method.

**TABLE 1:** Amount of fruits processed by leading Sri Lankan fruit processing companies and average percentages of fruit processing wastes.

Fruit waste	Amount of processing fruits (MT)				Total (MT)	Waste type	Waste %	Total waste %
	A*	B*	C*	D*				
Pineapple	493.04	1594.83	217.99	650.00	2955.87	Peel	40.12 ± 4.00	69.98 ± 5.86
						Bagasse	17.66 ± 2.08	
						Stem and crown	12.20 ± 2.00	
Papaya	40.85	686.52	245.34	115.00	1087.72	Peel	37.10 ± 3.00	48.41 ± 4.58
						Seed	11.31 ± 1.73	
Mango	505.91	60.50	589.26	226.00	1381.67	Peel	22.24 ± 4.00	50.07 ± 5.66
						Seed	27.83 ± 2.65	
Passion fruit	445.57	1.50	NP	350.00	797.07	Peel	52.50 ± 4.30	61.21 ± 3.66
						Seed	8.75 ± 0.64	
Wood apple	223.44	NP	185.72	200.00	609.16	Shell, seed and fiber	unseparated	50.41 ± 7.78
Ash gourd	163.02	NP	NP	400.00	563.02	Peel and seed	unseparated	46.57 ± 8.48
Lime	81.30	117.12	17.82	125.00	341.24	Peel and seed	unseparated	62.32 ± 8.72

*Note*: A, B, C, D are Local companies in Sri Lanka. Values are means ± standard deviation of triplicate measurements.

Abbreviations: NP, Not processed during the survey period.

Among the fruits evaluated, pineapple generated the highest proportion of waste, accounting for 69.98 ± 5.86% of the initial fruit weight. The major waste fractions of pineapple were peel (40.12% ± 4.00%), bagasse (17.66% ± 2.08%), and stem and crown (12.20% ± 2.00%) (Table 1). Lime (62.32% ± 8.72%) and passion fruit (61.21% ± 3.66%) also exhibited high total waste percentages. In mango and papaya processing, total waste accounted for 50.07% ± 5.66% and 48.41% ± 4.58% of the fresh fruit weight, respectively. Peels constituted the predominant waste fraction for passion fruit (52.50% ± 4.30%), pineapple (40.12% ± 4.00%), papaya (37.10% ± 3.00%), and mango (22.24% ± 4.00%). In mango processing, seed kernels represented a substantial proportion of waste (27.83% ± 2.65% of fresh fruit weight). For wood apple, ash gourd, and lime, individual waste fractions were not separated during industrial processing; therefore, only total waste percentages were recorded.

These findings are consistent with previous reports indicating that the mango peel and seed together constitute approximately 45% of the fresh fruit weight (Gupta and Joshi 2000; Mitra et al. 2013). Overall, the results demonstrate that significant quantities of fruit processing wastes are generated in Sri Lanka, highlighting their potential availability as raw materials for value‐added applications in the food industry.

### 3.2 Nutritional Composition of Fruit Waste

#### 3.2.1 Crude Protein

Fruit waste samples generated from commonly processed fruits in Sri Lanka were analyzed for proximate composition, including crude protein, crude fat, crude fiber, and ash (Table 2). The crude protein content of fruit waste samples ranged from 0.50%–25.94% on a dry weight basis. The highest protein content was observed in papaya seed (25.94%), followed by watermelon seed (17.59%), papaya peel (13.43%), and watermelon peel (11.11%). Banana peel also exhibited a moderate protein content (6.74%). Most other waste fractions contained less than 5% of crude protein (Table 2).

**TABLE 2:** Proximate composition of industrial fruit wastes in Sri Lanka.

Fruit waste types	Moisture%	Ash %	Crude Protein %	Crude Fat %	Crude Fiber %	Carbohydrate %
Watermelon seed	1.41 ± 0.06	2.21 ± 0.10^i^	17.59 ± 0.16^b^	25.40 ± 1.13^b^	43.10 ± 0.88^a^	10.29
Watermelon peel	7.29 ± 2.02	6.53 ± 0.14^de^	11.11 ± 0.52^d^	1.60 ± 1.03^gh^	30.67 ± 0.30^e^	42.80
Mango seed kernel	4.03 ± 0.19	1.86 ± 0.23^i^	5.94 ± 0.86^ef^	8.10 ± 0.14^d^	13.04 ± 0.84^i^	67.03
Mango peel	7.50 ± 0.40	2.00 ± 0.10^i^	3.10 ± 0.40^g^	4.43 ± 0.11^f^	18.78 ± 0.05^gh^	64.19
Banana peel	6.26 ± 0.36	13.92 ± 0.01^a^	6.74 ± 0.06^e^	8.19 ± 0.14^d^	18.46 ± 0.04^gh^	46.43
Papaya seed	5.47 ± 0.26	6.92 ± 0.56^d^	25.94 ± 0.02^a^	9.91 ± 0.05^c^	38.68 ± 0.48^c^	13.08
Papaya peel	9.25 ± 0.11	8.80 ± 0.72^c^	13.43 ± 0.77^c^	2.76 ± 0.91^g^	12.97 ± 0.14^i^	47.30
Pineapple bagasse	6.66 ± 0.04	2.34 ± 0.02^i^	4.10 ± 0.02^g^	2.49 ± 0.43^g^	29.20 ± 0.78^e^	55.20
Ash gourd seed	3.53 ± 0.30	3.53 ± 0.04^h^	1.63 ± 0.006^h^	28.16 ± 0.28^a^	40.83 ± 0.53^b^	22.32
Ash gourd peel	5.77 ± 0.07	9.66 ± 0.11^b^	1.29 ± 0.00^hi^	2.27 ± 0.02^g^	25.96 ± 0.34^e^	55.05
Lime peel	10.46 ± 2.10	5.76 ± 0.08^f^	0.58 ± 0.01^hi^	6.87 ± 0.04^df^	19.34 ± 0.10^g^	56.99
Passion fruit peel	7.13 ± 0.03	6.05 ± 0.10^ef^	0.5 ± 0.01^i^	0.36 ± 0.01^h^	34.21 ± 1.02^d^	51.75
Pineapple peel	6.37 ± 0.09	4.33 ± 0.04^g^	5.39 ± 0.04^f^	5.35 ± 0.05^ef^	17.06 ± 0.08^h^	61.50

*Note*: Values are expressed in dry basis as mean ± Standard deviation (*n* = 13), Means with different superscript letters in individual column are significantly (*p* < 0.05) different from each other (turkey's test).

In general, seeds exhibited higher crude protein contents than peels of the same fruit, as seeds are nutrient‐dense tissues, whereas peels are primarily composed of structural carbohydrates and therefore contain lower protein levels. Within peel wastes, considerable variability was observed, with passion fruit peel (0.50%) and lime peel (0.58%) exhibiting particularly low protein contents. Crude protein values in selected fruit peels, including papaya, watermelon, banana, mango, and pineapple, have been reported to range from 5.00% to 18.06% (Romelle et al. 2016), consistent with the present findings. Mango seed kernels in the current study contained 5.94%–27.83% protein, which is comparable to values reported for Kenyan mango seed kernels (6.74%–9.20%) (Mutua et al. 2017).

Recent advances in the valorization of fruit waste proteins highlight their potential as functional ingredients in food applications. Mango seed kernel proteins are rich in essential amino acids, particularly leucine and arginine, and exhibit high foaming and emulsifying capacities (Abdalla et al. 2007; Ramírez‐Maganda et al. 2015). Similarly, banana peel proteins can be fractionated into albumin, globulin, prolamin, and glutelin, with albumin showing high water‐ and oil‐holding capacities and emulsion potential, while glutelin demonstrates superior foaming capacity and dispersibility (Deb et al. 2022). These findings indicate that proteins derived from fruit waste possess valuable functional properties that can be exploited to develop innovative food products.

#### 3.2.2 Crude Fat

Crude fat content of fruit wastes ranged from 0.36% in passion fruit peel to 28.16% in ash gourd seed (Table 2). Generally, fruit seeds contained higher fat levels (8.10%–28.16%) than peels, reflecting their role as energy‐dense storage tissues. Plant‐derived fats are rich in beneficial fatty acids and can contribute to both nutritional and functional properties. For example, papaya seed oil has been reported to support digestive health and to be used as a food seasoning (Okeniyi et al. 2007; Lee et al. 2011). Mango‐derived fats are also rich in palmitic, stearic, oleic, and linoleic acids, offering additional value for functional food applications. Mango seed kernel oil, containing 9.84%–18.0% fat, is blended with other vegetable oils to produce cocoa butter alternatives, enhancing the sensory and nutraceutical properties of chocolate and other food products (Choudhary et al. 2023; Abbas et al. 2023).

Industrial fruit processing waste consisted primarily of peels, which generally have lower fat content than seeds (0.36%–4.43%), except for banana peel (8.19%) (Table 2). The lipid fraction of fruit peels is largely composed of nonpolar compounds, such as carotenoids, skin waxes, and latex, which contribute to their high fat content. In the present study, mango and papaya peels contained 4.43% and 2.76% crude fat, respectively, which are consistent with previously reported values (Omutumbga et al. 2012; Okai et al. 2010). Banana peel fat in our study (8.19%) is slightly higher than that reported by Hikal et al. (2022) (5.93% ± 0.13%) and aligns with Munguti et al. (2006) (7.9%). The lipid fraction of banana peel is particularly rich in polyunsaturated fatty acids, mainly linoleic acid and α‐linolenic acid. These findings indicate that fruit peel‐derived lipids may have potential for incorporation into food formulations.

#### 3.2.3 Crude Fiber

Table 2 shows that fruit wastes are rich sources of dietary fiber, with values ranging from 13.04% in mango seed kernel to 43.10% in watermelon seed. High fiber contents were also observed in papaya peel (12.97%), mango peel (18.78%), pineapple peel (17.06%), and banana peel (18.46%). These findings are consistent with those reported by Romelle et al. (2016), who documented comparable fiber levels in papaya peel (12.16%), mango peel (15.43%), pineapple peel (14.80%), and banana peel (11.81%). Dietary fiber derived from fruit waste can be used as a low‐calorie bulk ingredient in food formulations or as a raw material for fiber extraction (Chau and Huang 2004; Rohm et al. 2015). In addition, dietary fiber present in fruit wastes exhibits prebiotic properties and contributes to improved digestive health (Holscher 2017).

Recent studies have demonstrated the successful incorporation of fruit peel flours into food products to enhance fiber content and functional properties. For example, banana and mango peels have been explored as functional ingredients in baked products due to their high fiber contents (Ismail et al. 2024), while melon peel flour has been shown to improve the nutritional quality of bakery products without compromising sensory acceptability (Silva et al. 2024). Overall, the present study highlights industrial fruit waste as an abundant and underutilized resource for the development of value‐added, fiber‐rich food products such as bakery items and dairy‐based formulations, while simultaneously contributing to waste reduction and environmental sustainability.

#### 3.2.4 Carbohydrates

Carbohydrate contents of the analyzed fruit waste ranged from 10.29% to 67.03% (Table 2), reflecting substantial variation among waste types. Higher carbohydrate levels were associated with wastes having lower fat and protein contents, whereas wastes rich in fat and fiber, such as watermelon and ash gourd seeds, showed lower carbohydrate values. Since carbohydrate content was calculated by difference, elevated levels of crude fiber, fat, and protein directly contributed to reduced carbohydrate percentages in certain samples. These findings indicate that several fruit wastes constitute carbohydrate‐rich matrices that could be exploited for fermentation‐based applications, such as wine and vinegar production, as well as for the development of natural sweeteners or thickening agents in food formulations (Ismail et al. 2024; Silva et al. 2024). Therefore, the utilization of fruit waste within a scientific approach would be feasible to meet consumers' requirements for natural, functional, healthy food.

### 3.3 Mineral and Heavy Metal Composition

The concentrations of essential minerals (Na, Mg, K, Ca, Zn, and Cu) in industrial fruit wastes are presented in Table 3. Potassium was the most abundant mineral across all samples, ranging from 7.97 to 65.86 mg/g, with particularly high levels in banana and watermelon peels. Both papaya and ash gourd seeds and peels exhibited appreciable calcium content (>12 mg/g), indicating their potential contribution to dietary mineral intake. Magnesium levels were notably higher in ash gourd seed (4.27 mg/g) and papaya seed (3.67 mg/g). In addition, zinc and copper were present at elevated concentrations in several fruit wastes, especially papaya seed, watermelon seed, and ash gourd seed, highlighting the nutritional relevance of these underutilized by‐products as sources of essential trace elements.

**TABLE 3:** Mineral composition of industrial fruit waste samples (mg/g of dried sample).

Waste types	Na	Mg	K	Ca	Zn	Cu
Ash gourd seed	0.38	4.27	16.89	1.74	43.81	28.99
Ash gourd peel	0.29	3.35	22.65	12.43	24.35	8.81
Watermelon seed	0.19	1.71	9.49	1.08	46.02	15.69
Watermelon peel	1.50	1.43	65.68	6.95	30.89	7.68
Mango seed kernel	0.25	0.64	7.97	0.99	0.40	8.78
Mango peel	0.16	1.13	20.96	7.07	15.76	9.91
Papaya peel	2.57	1.98	38.85	14.31	19.64	2.21
Papaya seed	0.83	3.67	35.96	12.77	53.40	9.71
Pineapple peel	0.38	0.18	34.18	6.44	42.32	7.93
Pineapple bagasse	1.01	0.43	13.00	1.10	26.97	7.02
Banana peel	0.33	1.38	65.86	5.88	13.97	2.40
Passion peel	2.92	1.81	49.37	10.96	22.59	8.21
Lime peel	0.43	1.10	39.00	27.96	20.71	24.25

Abbreviations: Na, sodium; Mg, magnesium; Ca, calcium; K, potassium; Zn, zinc; Cu, copper.

The concentrations of heavy metals, arsenic (As), cadmium (Cd), mercury (Hg), and lead (Pb) in industrial fruit processing wastes are presented in Table 4. All samples contained As, Cd, and Hg at levels below the maximum permissible limits established by the Codex Alimentarius (Cd: 0.05 mg/kg; As: 0.50 mg/kg; Hg: 0.50 mg/kg), indicating low risk associated with these elements. Although Cd concentrations in passion fruit peel, pineapple bagasse, and watermelon peel were relatively higher compared to other samples, they remained within acceptable limits. In contrast, Pb concentrations exceeded the recommended limit (0.10 mg/kg) in several samples, particularly lime peel, passion fruit peel, and ash gourd seed. These findings highlight the importance of implementing good agricultural and processing practices throughout the food value chain to minimize heavy metal contamination in fruit processing by‐products.

**TABLE 4:** Heavy metal content of industrial fruit waste samples (mg/kg of dried sample).

Type of fruits	As	Cd	Hg	Pb
Ash gourd seed	0.017	0.022	0.007	2.954
Ash gourd peel	0.010	0.005	0.016	0.131
Watermelon seed	0.005	0.010	0.045	1.036
Watermelon peel	0.003	0.061	0.010	0.297
Mango seed kernel	0.002	0.010	0.001	0.623
Mango peel	0.003	0.010	0.002	0.328
Papaya peel	0.081	0.017	0.006	0.125
Papaya seed	0.036	0.015	0.001	0.024
Pineapple peel	0.064	0.020	0.006	0.001
Pineapple bagasse	0.032	0.116	0.001	0.016
Banana peel	0.009	0.011	0.000	0.170
Passion peel	0.013	0.134	0.012	4.080
Lime peel	0.066	0.016	0.026	8.008

Abbreviations: As, arsenic; Cd, cadmium; Hg, mercury; Pb, lead.

### 3.4 Antioxidant Capacity of Fruit Wastes

#### 3.4.1 Total Phenolic Content (TPC)

Phytochemicals, including phenolics, contribute to the antioxidative activity of fruit wastes. In the present study, the highest total phenolic content (mg Gallic Acid Equivalent/g), estimated by reactivity toward the Folin‐Ciocalteu reagent, was observed in mango seed kernel (9.47 ± 0.21), followed by mango peel (8.64 ± 0.06), lime peel (8.62 ± 0.25), and papaya peel (8.20 ± 0.10), which were significantly higher (*p* < 0.05) compared to other waste types analyzed (Table 5). These results are comparable to previous reports showing that methanolic extracts of mango peel and seed exhibit substantial phenolic content (Ajila et al. 2010; Nguyen et al. 2019). The variation in TPC among fruit wastes reflects the diverse nature of the source fruits and the type of residue generated.

**TABLE 5:** Total phenolic content (TPC), total flavonoid content (TFC), DPPH radical scavenging capacity and total antioxidant capacity (TAC) of industrial fruit wastes (dry basis).

Sample type	TPC (mg GAE/g)	TFC (mg RE/g)	IC_50_ (mg AAE/mL)	TAC (mg AAE/g)
Papaya seed	2.13 ± 0.08^i^	5.38 ± 0.27^e^	31.72 ± 0.09^b^	1.85 ± 0.1^j^
Passion peel	3.89 ± 0.0^h^	1.38 ± 0.07^h^	20.93 ± 0.12^d^	6.15 ± 0.28^h^
Pineapple bagasse	5.75 ± 0.11^e^	0.72 ± 0.09^h^	10.18 ± 0.33^h^	18.10 ± 0.16^d^
Pineapple peel	5.98 ± 0.11^e^	2.17 ± 0.13^g^	18.85 ± 0.21^f^	14.71 ± 0.19^e^
Lime peel	8.62 ± 0.25^b^	4.32 ± 0.11^f^	5.89 ± 0.07^h^	19.2 ± 0.63^c^
Papaya peel	8.20 ± 0.10^c^	2.58 ± 0.07^g^	7.56 ± 0.11^j^	23.93 ± 0.49^b^
Mango seed kernel	9.47 ± 0.21^a^	21.82 ± 0.51^a^	0.53 ± 0.06^m^	20.27 ± 0.57^c^
Mango peel	8.64 ± 0.06^b^	12.64 ± 0.22^c^	1.66 ± 0.11^l^	25.54 ± 0.66^a^
Banana peel	2.33 ± 0.09^i^	8.79 ± 0.29^d^	27.23 ± 0.13^c^	10.87 ± 0.58^g^
Watermelon seed	4.81 ± 0.11^f^	13.10 ± 0.03^c^	15.66 ± 0.16^g^	3.24 ± 0.14^i^
Watermelon peel	7.21 ± 0.17^d^	3.65 ± 0.07^f^	9.47 ± 0.09^i^	18.97 ± 0.24^d^
Ash gourd seed	4.40 ± 0.08^g^	17.64 ± 0.47^b^	54.18 ± 0.26^a^	2.95 ± 0.23^ij^
Ash gourd peel	5.13 ± 0.14^f^	5.05 ± 0.16^h^	19.74 ± 0.22^e^	12.48 ± 0.35^e^

*Note*: Values are expressed on a dry basis as mean ± standard deviation (*n* = 3). Means with different superscript letters in individual columns are significantly (*p* < 0.05) different from each other (turkey's test).

Abbreviations: AAE, acetic acid equivalent respectively; AAE, ascorbic acid equivalent;

GAE, gallic acid equivalent; RUE, rutin equivalent.

#### 3.4.2 Total Flavonoid Content (TFC)

Flavonoid contents of fruit wastes were estimated using the aluminum chloride colorimetric method and expressed as mg Rutin Equivalents (RE) per g of dry weight (Table 5). Mango seed kernel (21.82 ± 0.51), ash gourd seed (17.64 ± 0.47), watermelon seed (13.10 ± 0.03), and mango peel (12.64 ± 0.22) exhibited the highest flavonoid contents. Previous studies have identified flavonoids such as digallic acid, procyanidin A2, quercetin hexoside, quercetin, kaempferol, galloylglucoside, mangiferin, and allergic acid in mango peel and seed, contributing to cellular antioxidant defenses and maintenance of physiological functions (Nguyen et al. 2019; Joshi et al. 2012).

The types and concentrations of antioxidants in different fruit waste sources vary significantly, reflecting the diverse nature of these materials. Similar trends have been observed in other fruit by‐products, where antioxidant potential depends on the fruit type and the specific residue (Marin‐Tinoco et al. 2023; Frasão et al. 2021). The apparent higher TFC values compared to TPC in some samples can be attributed to the use of rutin as the standard for flavonoids and gallic acid as the standard for phenolics, as well as differences in the analytical methods employed for their estimation. Moreover, the differences between TPC and TFC values also suggest the presence of non‐flavonoid phenolic compounds and other reducing substances (such as ascorbic acid, sugars, and organic acids) in the extracts, which may interfere with the Folin–Ciocalteu assay and contribute to variations in TPC measurements.

#### 3.4.3 DPPH Radical Scavenging Activity

In the DPPH assay, lower IC_50_ values indicate greater antioxidant activity. In the present study, mango seed kernel (0.53 mg/mL) and mango peel (1.66 mg/mL) exhibited the strongest DPPH radical scavenging capacity, followed by lime peel (5.89 mg/mL) and papaya peel (7.56 mg/mL) (Table 5). These results correspond well with the higher total phenolic and flavonoid contents observed in these samples, supporting the role of phenolic compounds as major contributors to free‐radical scavenging activity. Variations in IC_50_ values among fruit wastes can be attributed to differences in antioxidant composition, fruit type, and processing conditions (Marin‐Tinoco et al. 2023; Frasão et al. 2021). Similar observations have been reported in recent studies highlighting mango and banana processing by‐products as rich sources of antioxidants with strong radical scavenging potential and applicability in functional food systems (Ismail et al. 2024; Silva et al. 2024).

#### 3.4.4 Total Antioxidant Capacity (TAC)

The total antioxidant capacity (TAC) of fruit waste samples, expressed as mg ascorbic acid equivalents (AAE) per g of dry weight, varied widely among the analyzed wastes, ranging from 1.85 to 25.54 mg AAE/g (Table 5). Mango peel exhibited the highest TAC value (25.54 mg AAE/g), followed by papaya peel (23.93 mg AAE/g), mango seed kernel (20.27 mg AAE/g), lime peel (19.20 mg AAE/g), and watermelon peel (18.97 mg AAE/g). In contrast, lower TAC values were observed in papaya seed (1.85 mg AAE/g) and ash gourd seed (2.95 mg AAE/g), indicating comparatively weaker total antioxidant potential.

The high TAC values observed in mango peel, papaya peel, and mango seed kernel are consistent with their elevated total phenolic and flavonoid contents, suggesting that these compounds contribute substantially to the overall antioxidant capacity. Similar trends, where peels and seed kernels exhibit higher antioxidant potential than other waste fractions, have been reported in recent studies on fruit by‐products (Ismail et al. 2024; Silva et al. 2024).

In this study, antioxidant potential was assessed using four complementary assays (TPC, TFC, DPPH, and TAC), which together provide a comprehensive evaluation of antioxidant behavior. Variations between assays and differences compared with previous studies may be attributed to cultivar differences, ripening stage, seasonal variation, agro‐ecological conditions, and methodological differences in extraction and analysis. Overall, the results demonstrate that selected industrial fruit wastes, particularly mango and papaya processing by‐products, represent promising sources of natural antioxidants for potential functional food applications.

## 4 Conclusion and Future Perspectives

The present study demonstrates that a substantial proportion of fruits processed in Sri Lanka is converted into waste, mainly comprising peels, seeds, and bagasse. These by‐products were shown to contain appreciable levels of macronutrients, essential minerals, and bioactive compounds, with marked variation across fruit types and waste fractions. Seeds generally exhibited higher protein and fat contents, while peels and bagasse were richer in dietary fiber and carbohydrates. Most fruit wastes also displayed notable antioxidant potential, particularly mango peel, mango seed kernel, papaya peel, and lime peel, as evidenced by their phenolic and flavonoid contents, DPPH radical scavenging activity, and total antioxidant capacity. Heavy metal levels were largely within internationally acceptable safety limits, supporting their potential for food‐related applications under proper processing conditions.

From a future perspective, these findings highlight the feasibility of valorizing industrial fruit waste as a sustainable source of functional ingredients for food and nutraceutical applications. Further studies should focus on optimizing extraction techniques, characterizing individual bioactive compounds, evaluating functional and techno‐functional properties in food systems, and assessing bioavailability and safety through in vivo studies. The integration of fruit waste valorization into the food processing chain could help reduce waste, promote environmental sustainability, and develop value‐added functional foods.

## Author Contributions

**Chamila Vinodanee Liyanage Jayasinghe**: conceptualization, editing and reviewing, supervision, **Jayasundara Mudiyanselage Lakshan Randika Bandara**: methodology, data curation, original manuscript drafting, **Rankothge Chamodini Nisansala Thilakarathna**: manuscript writing, editing and reviewing, supervision

## Funding

We greatly acknowledge the financial support provided by the National Science Foundation of Sri Lanka (NSF) (Research Grant; NSF/ RG/2017/AG/02).

## Conflicts of Interest

The authors declare no conflicts of interest.
